# Cellular and Pharmacological Targets to Induce Coronary Arteriogenesis

**DOI:** 10.2174/1573403X113099990003

**Published:** 2014-02

**Authors:** Maurits R. Hollander, Anton J.G. Horrevoets, Niels van Royen

**Affiliations:** a Department of Cardiology, VU University Medical Center, Amsterdam, The Netherlands;; b Department of Molecular Cell Biology and Immunology, VU University Medical Center, Amsterdam, the Netherlands

**Keywords:** Arteriogenesis, CAD, collateral, endothelium, growth factors, proliferation, SMC.

## Abstract

The formation of collateral vessels (arteriogenesis) to sustain perfusion in ischemic tissue is native to the body and can compensate for coronary stenosis. However, arteriogenesis is a complex process and is dependent on many different factors. Although animal studies on collateral formation and stimulation show promising data, clinical trials have failed to replicate these results. Further research to the exact mechanisms is needed in order to develop a pharmalogical stimulant. This review gives an overview of recent data in the field of arteriogenesis.

## INTRODUCTION

Obstructive coronary artery disease brings along morbidity and mortality in millions of patients worldwide [1]. Despite the success of techniques like retrograde revascularization by coronary artery bypass grafting and antegrade revascularization by percutaneous coronary intervention (PCI), these forms of treatment are not suited for all patients due to complexity of the lesions or co-morbidity and high surgical risks. Therefore new alternative treatment options are warranted to alleviate symptoms and improve survival and quality of life in these estimated 20% of patients without revascularization options. One of the body’s physiologic mechanisms to maintain tissue perfusion is the growth of collateral vessels. Due to an increasing gradient in the presence of a arterial stenosis, the caliber of pre-existing collateral vessel is increased, This process is known as arteriogenesis and is in concept the prototype of retrograde vascularisation using natural bypasses already present in myocardial tissue [2]. This potent process is capable to largely compensate for perfusion deficits in coronary artery disease (CAD) and its mechanisms have been under extensive research in the past decades. Coronary collaterals are already prevalent in some extent in every person [3]. Interestingly, only about one third of the patients develops a fully functional collateral circulation and therefore stimulation of arteriogenesis is considered a promising approach for CAD treatment. This review focuses on the known molecular mechanisms of arteriogenesis and gives an outlook to potential future clinical applications. 

## PRE-EXISTING COLLATERAL ARTERIES

Cardiac muscle is perfused via the coronary arteries, mainly during diastole. Coronaries branch and vessel diameter declines until a capillary network is formed. The three coronaries: the right coronary artery (RCA), left anterior descending artery (LAD) and the left circumflex artery (Cx) all have their own perfusion territory, but these territories do partly overlap, mainly in the microvasculature. Here some of the small arteries deriving from different coronaries connect, forming arterial-arterial anastomoses and thus, a collateral circulation [4]. As stated, these connections are predominantly found in the intramyocardial microvasculature and therefore, most of the collateral arteries can be found deep within the cardiac muscle. Such collateral connections are present in normal cardiac tissue, although vessel diameter is small and flow is minimal. The presence of these collateral vessels was shown by Fulton more than half a century ago [5]. A more recent study on the presence of collateral vessels in patients without CAD showed not only the presence but also the functionality of preformed collateral arteries in non-ischaemic heart tissue [3, 6]. In normal healthy coronary arteries, blood pressure is nearly equal in the different vascular territories and therefore the pressure gradient over the collateral connections is very low, preventing appreciable flow. If coronary vessel diameter reduces due to plaque formation, post-stenotic blood pressure drops. The difference in pressure creates a gradient over the collateral arteries and thereby enhances flow. This process occurs spontaneously and is a natural mechanism to reduce hypoperfusion. Giving the right conditions, the relatively small collaterals can grow sufficiently to sustain adequate oxygenation to the affected myocardium at risk of ischemia. The extent of collateral flow can be determined by the collateral flow index (CFI) [7]. The favorable effects of arteriogenesis have been shown in a study of 845 patients of which 739 patients had stable angina. After determining CFI and collecting follow-up information for 10 years, it was concluded that the presence of a well-devolved collateral network was related to a 25% lower all-cause mortality rate [8]. A recent meta-analysis of a total of 12 studies showed that patients with a high collateralization even have a 36% reduced mortality risk compared to patients with low collateralization [9]. Therefore, adequate stimulation of arteriogenesis can have a major impact on current treatment of patients with chronic ischemic heart disease.

## HYPERPERFUSION/SHEAR STRESS

The progressing narrowing of a vessel causes a higher resistance in the affected artery and diminishes the pressure gradient. Thereby, collateral connections in these territories will receive increasing volumes of flow. To begin with, the pre-existing connections have a relative small diameter and the sudden increase in flow causes an ambiguous effect on the vessel wall. The friction created by the altered blood flow puts a longitudinal strain on the vessel wall, called fluid shear stress (FSS). This must not be confused with circumferential wall stress which is dependent on the intraluminar pressure and wall thickness. FSS is a relative weak force compared to the circumferential wall stress and effects predominantly the endothothelium. Research in pigs, dogs and rabbits have shown that FSS is the molding force behind arteriogenesis [10-12]. It is believed that an increase in shear stress initiates the cascade of events that eventually leads to adequately functioning collateral arteries. This mechanism is not only seen in peripheral tissue but also in the heart and brain [13, 14]. Measuring the specific effect of FSS is rather difficult from a methodical point of view. Pre-existing collaterals are redundant, small in diameter and usually embedded deep within myocardial tissue. Pipp *et al*. have managed to demonstrate the importance of FSS in a porcine model of FSS [15]. By creating an aterio-venous anastomose in the hind limb, they managed to reach extreme levels of FSS, so that it’s specific effect could be measured. Others, have shown that FSS modulates the endothelium, elongating and stretching the cells [16,17]. The increase in flow activates the EC’s causing them to proliferate and facilitate luminar expansion. This remodeling is accompanied with the release of several different proteins. For optimal effect the shear stress must have pulsatile character, as could be expected is arteries [18,19]. This is also shown for circumferential stress [20]. Although the role of shear stress and shear stress responsive elements (SSRE) seems paramount, others have claimed that also the circumferential wall stress plays a role in the development of a collateral circulation [21]. For example, the expression of platelet endothelial cell adhesion molecule (PECAM)-1 is unregulated by increased wall stress, acting as a mechanosensor for endothelial cells. Interestingly it was recently shown that PECAM-1 also regulates the diameter of pre-existing collaterals [22]. 

## MONOCYTE RECUITMENT

Monocytes play a crucial role in arteriogenesis and the concentration of circulating monocytes is directly linked to the degree of vessel proliferation after ligation [12, 23, 24]. Monocytes act in a local fashion and need to be attracted to the site of interest in order to sort an arteriogenic effect. The FSS-activated vascular endothelium excretes different adhesion molecules to recruit circulating monocytes. One of the most studied molecules is monocyte chemotactic protein 1 (MCP-1, also known as CCL-2). Intracellular adhesion molecule-1 (ICAM-1) and vascular cell adhesion molecule-1 (VCAM-1) also play an important role in this mechanism. The total cascade leading to the appearance of monocytes in the perivascular space consists of monocyte emigration of the bone marrow, chemo-attraction to the vessel wall, rolling along and adhesion to the luminar side of the endothelium and finally transmigration into the perivascular space. 

The sudden increase in FSS causes the endothelium to produce MCP-1 [25]. Treatment with MCP-1 leads to an increased arteriogenic response in different animal models [26-28]. MCP-1 treated animals showed not only increased collateral perfusion, but also increased numbers of monocytes/macrophages around arterial vessels, showing monocyte involvement in the remodeling process. Peak numbers of monocytes/macrophages are seen around 3 days after ligation, after which a gradual decline is seen. In MCP-1 -/- mice perfusion restoration is diminished after ligation [29]. Although MCP-1 is widely studied, genetic research of femoral ligated mice showed an involvement of several other chemokines, including CCL3 (MIP-1α) and CCL7 (MCP-3), but also CXCL9 and 10 [30].

## MACROPHAGE POLARIZATION

**Fig. (1):**
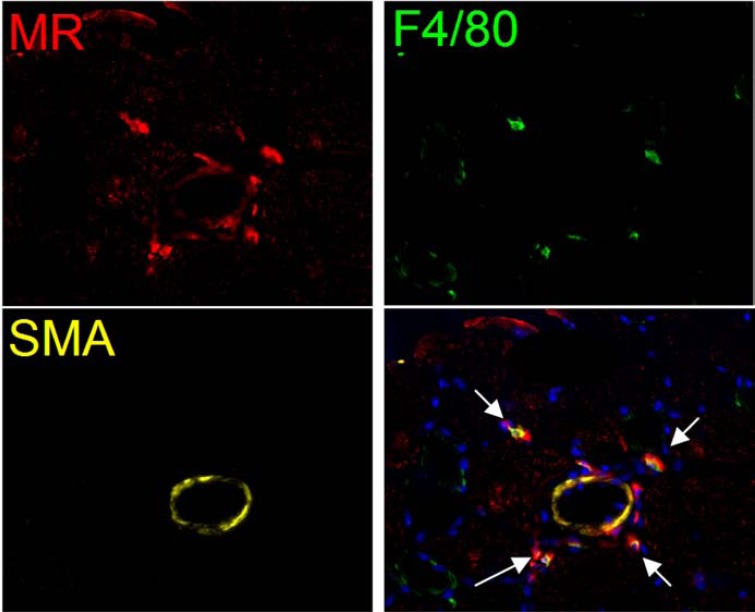
Murine hindlimb muscle tissue. Immunofluorescent staining showing manose receptor (red) positive and F4/80 (green) positive cells (M2 macrophages) around collateral arteries containing smooth muscle actin (yellow). Nuclei are stained blue. 20x magnification.

After adhering to the endothelium, monocytes transmigrate into the perivascular space were they become macrophages. The adherence and presence of monocytes in and around collateral vessels has been demonstrated histologically by Schaper *et al.* in 1976 [31]. The role and function of these cells in collateral formation have since then been examined thoroughly. Many animal studies have demonstrated that inducing arteriogenesis is accompanied by an influx of monocytes/macrophages. Comparable to T-cells, also macrophages can polarize and at least two phenotypes can be distinguished in humans. M1-type macrophages, which play a role in inflammation and pathogen resistance and M2-type macrophages which play a role in wound healing and vascular proliferation (Fig. **1**). It is classically believed that Interferon(IFN)-γ shifts cells to the M1 phenotype and that IL-4 and IL-13 are responsible for the M2-differentiation. Nowadays it is known that there are several other factors capable of M2 polarisation such as Il-10 and IL-33 [32, 33]. The polarisation translates to a different gene set expressed by M1 and M2 macrophages. Consequently, different chemokines are produced [34]. 

As shown by Takeda *et al*., increasing the levels of M2 macrophages by shifting the M1:M2 balance in Phd haplodeficient mice, induces the formation of collateral vessels and reduces hypoxic stress after femoral artery ligation. By transplanting genetically modified bone marrow (from Phd2 haplodeficient mice who predominantly produce M2 macrophages) into wild type mice they confirmed the key role of bone marrow derived cells in collateralization. Moreover, at baseline the Phd2 haplodeficient animals showed significant higher levels of perivascular M2 macrophages. This suggests that not only the attracted monocytes, but also tissue-resident macrophages (in particular M2’s) are essential for effective collateral vessel growth [35]. This spacial distribution has recently been confirmed by histological analysis, showing a shift in local distribution of subtypes during arteriogenesis. It is demonstrated that M2 macrophages do reside in the perivascular space in non-occluded tissue, although low in number. After ligation these numbers rise rapidly, reaching maximal levels at day 3 [36]. Jenkins *et al*. even claim that M2 macrophage accumulation is independent of circulating monocytes and is only driven by local proliferation. They confirm that it is the IL-4 protein, normally produced by T-helper-2 cells, that induces the shift to M2 [37, 38]. This data suggests that the perivascular population of macrophages during arteriogenesis has more than one origin. The potential to proliferate tissue resident cells opens new ways to overcome the hurdle of locally attracting sufficient amounts of monocytes to stimulate collateral growth and possibly suppress systemic effects, like atherosclerosis. 

## VASCULAR PROGENITOR CELLS

During arteriogenesis, macrophage-derived factors induce the proliferation of resident EC’s and SMC’s, facilitating vessel expansion. Potentially, not only the resident vascular wall cells proliferate, but their precursors are also attracted to enhance arteriogenesis. In the endomyocardial microvasculature of patients with anginal complaints a type of mononuclear cells that express β-actin (β-MNC’s) are present. A recent study in a canine model showed that these cells can migrate to the perivascular space. Here they lose their β-actin and align with either SMC’s or EC. They adopt the phenotype of their parallel cells, acquiring either α-smooth muscle actin to become SMC’s or start expressing CD31 to become mature EC’s [39]. The fact that these cells can adopt the phenotypes of two different cell types that comprise the vascular wall makes them highly potential for supporting collateral growth. Others however have claimed that progenitor cells do not play a role in arteriogenesis by providing a source for new vascular wall cells but merely act in a paracrine way, providing some of the necessary growth factors [40].

## GROWTH FACTORS

## Vascular Smooth Muscle Cells And FGF’s

In order to grow into full functional arteries it is essential that the lumen and endothelium is surrounded by a layer of vascular smooth muscle cells (VSMC’s). This is indispensable for the capacity of arteries to control their diameter and thereby flow. One of the key steps in this process is the dedifferentiation of VSMC’s, in which the VSMC’s transform from heir contractile phenotype into a proliferative phenotype. This is mainly done through the loss of muscle specific structural proteins, predominantly desmin [41]. After this structural change, VSMC’s can proliferate and migrate to form the backbone of full functioning collateral arteries. In various animal models it was shown that fibroblast growth factors (FGF) are an important element in the arteriogenic response of VSMC’s. Of the four FGF’s, FGF-2 is the most studied [42]. However, following disappointing results with monotherapy now it has been put forth that growth factor combinations are more effective. Banquet *et al* used albumin-alginate microcapsules that sequentially release fibroblast growth factor-2 and hepatocyte growth factor in a coronary ligation model in rats, showing a multiple beneficial effect on cardiac remodeling and function[43]. This synergistic effect is also seen in the combination with granulocyte colony stimulating factor [44]. These animal studies suggest that adequate stimulation of arteriogenesis would require multiple compound therapy. In clinical experimental setting few studies on FGF-2 have yet been performed. The largest patients study determining the role of exogenous admitted FGF’s was the TRAFFIC study. 190 Patients with claudication were treaded with Recombinant fibroblast growth factor-2 (rFGF-2) or placebo. Peak walking time was significantly higher in the treatment group, suggesting better perfusion and arterial development [45]. To determine the effect of FGF-2 on the coronary circulation, patient with CAD were treated with single intracoronary injection of FGF-2 or placebo. Although there was a beneficial effect of the FGF-2 infusion on symptoms, nuclear perfusion imaging shown no significant effect [46]. Overall it can be concluded that the effects observed after exogenously applied bFGF were, at best, modest [47]. Currently, downstream targets of FGF-2 are studied for their potential to stimulate arteriogenesis. Activation of the mitogen-activated protein kinase (MEK)/extracellular signal-regulated kinase (Erk) pathway is essential for collateral atery growth [48]. This pathway has a large variety of signaling proteins and recently it has been shown that Rap-2 is significantly increased during arteriogenesis. Interestingly Rap2 stimulates VSMC migration, but not proliferation [49]. 

## GM-CSF and G-CSF

After promising data from animal studies, Seiler *et al.* showed that Granulocyte-macrophage colony-stimulating factor (GM-CSF) also induces arteriogenesis in humans [50]. However, subsequent studies examining the efficacy and safety of treatment of GM-CSF suggested that it might also induce acute coronary syndrome [51]. Therefore, GM-CSF is probably not a suitable candidate for coronary collateral growth promotion. Another colony-stimulating factor, granulocyte colony-stimulating factor (G-CSF), also promotes coronary collateral growth. In a controlled randomized trial it was demonstrated that therapy with G-CSF can salvage myocardial tissue in patients with chronic coronary artery disease [52]. Interestingly, unlike GM-CSF, data from a meta-analysis of G-CSF therapy in patients with acute MI.showed that treatment with G-CSF can be considered safe [53, 54] and thereby a potential candidate for clinical implementation. 

## VEGF

Vascular endothelial growth factor (VEGF) has been evaluated as a potential candidate for clinical stimulation of arteriogenesis [55]. Since its first discovery, several different isoforms of VEGF have been identified, VEGF-A being the most angiogenic. VEGF-A itself has also different isoforms and three different types of receptors bind to VEGF-A: VEGFR-1, VEGFR-2 and neuropilin-1 (NRP1), the first two being tyrosine kinase receptors [56]. Positive results in numerous animal models led to several small clinical studies that seemingly showed arteriogenic effects of VEGF in patients with either peripheral or coronary artery disease [57, 58]. Large studies however have failed to repeat these results. The randomized double-blind placebo controlled *VIVA* study showed no beneficial effect of a intracoronary infusion with VEGF, although anginal complaints and physical endurance did differ somewhat [59]. Currently, other components of the VEGF signaling-pathways are studied for their potential to stimulate arteriogenesis. The binding of VEGF-A to VEGF-R1 leads to the activation of second messenger pathways by binding adaptor molecules. One of these pathways that has recently been discovered by Fan *et al*. is the remodeling of actin by direct phosphorylation of profilin-1 (Pfn-1). The latter being a small protein abundantly present in endothelial cells that can mediate actin assembly, thereby promoting cell movement and thus, cell migration [60]. Interestingly, mice lacking Pfn-1 show no difference in vascular development in fetal mice, but impaired wound healing and arteriogenesis in a adult animals. This suggest a change in pathways activated by VEGF during life, although this has not been proven yet. 

## NEUREGULIN

Endothelial cells also produce neuregulins (NRG), a group of epidermal growth factor ligands that bind to erbB receptors. Previously it has been shown that the activation of erbB by NRG can induce angiogenesis [61]. Besides this, it has a protective role in the ischaemic damage of heart tissue *in vivo* [62]. This data suggests a more elaborate function of the NRG-erbB pathway tissue repair than only angiogenesis. Recently, the role of NRG-erbB during arteriogenesis was shown, using an endothelial selective NRG knockout model. Besides this the role of NRG on adhesion protein receptor integrins was examined. These molecules are needed for the proliferation, migration and differentiation of EC during angiogenesis. The absence of NRG impaired the activation of these integrins, further supporting the role of NRG in vascular growth. Additionally, administering human recombinant NRG to WT mice significantly improves flow recovery [61], suggesting a possible therapeutic agent to reduce ischaemic damage.

## PLATELET RICH PLASMA

As stated before, arteriogenesis is a native mechanism to the body and many of the described growth factors above can been found in platelets. Alpha granules found in platelets are known to carry multiple types of autologous growth factors, such as VEGF, FGF an HGF. To maximize the effect of these growth factors, platelet rich plasma (PRP) was developed years ago. PRP is known to have beneficial effects on native repair mechanisms [63]. Although potentially beneficial, PRP is derived from human blood and substantial differences are found in levels of growth factors, depending and donor characteristics and preparation method used [64, 65]. Most of the research on PRP has been done in the field of plastic surgery and soft tissue and wound repair. One of the problems for therapeutic arteriogenesis is to reach effective levels in targeted tissue. To overcome this problem several different carriers have been investigated. In the case of FGF-2, promising results were found using fragmin/protamine microparticles (F/G MP), showing strongly induced functional collateral vessels in the rabbit model of hindlimb ischemia [66]. Besides FGF-2, also HGF can be absorbed by the F/G MP’s to stimulate vascularisation, although this is only tested intradermally [67] . In a model by Fujita *et al*. the arteriogenic effect of F/G MPs together with PRP was tested. In an rabbit ischemia model animals were treated with either injections of placebo, PRP, F/G MPs or PRP containing F/G MPs. Results show a significant increase in number of collateral arteries and peripheral blood pressure in the PRP-F/G MP group compared to control, as well as treatment with only PRP or F/G MPs [68, 69]. Apparently, F/G MPs have an additive effect on the bioavailability of different proteins. By absorbing, the proteins are protected from heat and proteolysis and are released in a controlled manner [69]. A different approach to the local distribution of pharmacological stimulants is the coating of stents used in PCI. Drug eluting stents are nowadays clinically used world-wide and coating with pro-arteriogenic compounds seems to be promising [70], although pharmacological stimulation of collateral growth seems to be most beneficial for patients not suited for PCI.

## ADENOSINE AND OTHER VASODILATORS

The initiator of arteriogenesis is the increase in flow and thereby fluid shear stress (FSS). Therefore it seems logical that compounds that increase FSS, will induce arteriogenesis. In clinical practice there are several pharmalogical agents that induce vasodilatation. Adenosine is capable of inducing vasodilatation of the microvasculature and to stimulate angiogenesis [71]. However, it was also shown that absence of one the receptors of adenosine induces SMC proliferation under hypoxic condition in the lung [72]. Essential for the production of adenosine is CD73 or ecto-5′-nucleotidase. This cell surface molecule, expressed on endothelial cells, is crucial for the production of adenosine [73]. It is described that CD73-/- mice show an increased monocyte adhesion [74]. This suggests that adenosine could have an inhibitory effect on arteriogenesis. To study the effect of adenosine on arteriogenesis Böring *et al*. used mice lacking CD73 in an hindlimb ischaemia model. Using MRI and histology they demonstrate faster collateral formation in mutated mice. Furthermore, the R/L ratio of vessel diameter, wall thickness and surface area was significantly higher in the CD73-/- mice. Also, a significant difference in monocyte invasion was seen. All this supports the adverse effect of adenosine on arteriogenesis. Notably, the capillary density showed no difference between groups, indicating an absence of angiogenic effect. Nitrous oxide (NO) is also clinically used to temporarily increase vessel diameter and enhance flow. The increase in FSS up regulates the expression of endothelial Nitrous Oxide Synthase (eNOS). eNOS is known to induce neovascularisation but data on its arteriogenic effect are conflicting [75, 76]. Schirmer at al showed that lowering heart rate with ivabradine in mice induces an up regulation of eNOS and stimulates collateral artery growth [77]. A different study using a severe hindlimb ischemia model determined the effect of sodium nitrite, which augments tissue NO bioavailability. Using delayed sodium nitrate therapy they managed to rapidly increase ischemic limb arterial vessel diameter and branching. They conclude that treatment with sodium nitrate rapidly increases arteriogenesis in an NO-dependent manner [78]. It remains unclear whether NO directly influences cellular mechanisms or that the arteriogenic effect of NO is solely a result of the increase in FSS. 

## ENDOTHELIAL GAP JUNCTIONS

The intricate mechanism of arteriogenesis needs to be carefully coordinated in order to function properly. Complex signaling is needed to control adequate vascular remodeling. It is thought that gap junctions play an important communicative role in this process. These gap junctions are intercellular connections that mediate electrical and chemical coupling and are formed by different forms of connexin (Cx). The endothelium predominantly expresses two isoforms (of a total of 21) of connexin, namely Cx37 and Cx40. 

To investigate the role of these proteins Cx37 -/- and Cx40 -/- mice were created and post-ligation recovery was monitored in a severe hindlimb ischemia model. It was shown that Cx40 is required for perfusion restoration, where as Cx37 has an inhibitory effect on postischaemic limb survival [79]. In another study the effects Cx37 were examined in more detail, showing that Cx37 has an inhibitory effect on microvascular arteriogenesis as well on larger collateral artery formation [80]. The mechanisms underlying this effect are not entirely clear, but the influence of Cx37 on monocyte adhesion [81] may be of importance. Surprisingly, it has recently been found that shear stress, a known stimulator of arteriogenesis, increases expression of Cx37 in EC’s [81-83]. Cx37 also has a protective role in atherosclerotic plaque formation [84], making the pharmacological neutralization for arteriogenic goals less clinically applicable. 

## BRADYKININ SIGNALING

Bradykinin and its receptors (B1R and B2R) are known to have an effect on inflammation and neovascularisation. Data from previous research shows the influence of B1R and B2R on expression of the adhesion molecules ICAM-1 and VCAM-1, leukocyte recruitment [85] and NO production [86] among other vasoactive effects [87]. Although both receptors have impact, the contribution of B1R is considered to be more distinct. Until recently the effects of bradykinin signaling weren’t investigated. Hillmeister *et al.* have now showed that B1R acts as a positive modulator of arteriogenesis. In different animal models using B1R and B2R mutants the significant inhibitory effect of B1R on collateral formation was shown. Treatment with bradykinin receptor antibodies had the same effect, showing impaired perfusion restoration and diminished leukocyte-monocyte transmigration. also, they demonstrated that by administration of an B1R-agonist, cerebral arteriogenesis can be significantly increased. *In vitro*, B1R agonist effects on monocytes was tested using a human acute monocytic leukemia cell line monocyte migration assay. Results showed a strong increase in monocyte migration [88-90]. 

## NATURAL INHIBITORS OF ARTERIOGENESIS AS POTENTIAL NEW TARGETS 

Another way to stimulate VSMC proliferation is the blocking of inhibitory influences. By analyzing transciptomes of patients with either a well or a poor developed collateral circulation we identified a set of genes that correlated to collateral artery development in man. Interestingly, not the promoters of arteriogenesis showed a difference between groups, but the inhibitors were significantly altered. In the group of patients with the lowest CFI, also called the non-responders, these inhibitors were strongly expressed. On top of the list of differently expressed genes was interferon-beta (IFN-β) and several IFN-β related genes [91]. Subsequently, we found that administration of IFN-β in a murine femoral ligation model inhibits perfusion restoration, mainly by suppression of smooth muscle cell (SMC) proliferation. These results lead to believe that eliminating the effect of IFN-β could possibly have therapeutic potential. Other *in vivo* studies have shown that blocking IFN-β stimulates SMC proliferation and arteriogenesis. In mice lacking the IFN-β receptor-1 (IFNAR) perfusion is accelerated as compare to the control littermates. Although these results look promising it needs to be determined what other effects treatment with IFN-β or its antagonists contains. Similar to IFN-β, another protein has been revealed to be an inhibitor of arteriogenesis. Patients with a low arteriogenic response also showed significantly increased levels of galectin-2. The presence of Galectin-2 was seen on the surface of human monocytes, but could not be detected in the supernatant of cultured monocytes, suggesting an external adherence of galectin-2 onto monocytes, rather than excretion. Following these data, effects of exogenous Galectin-2 was tested in animal experiments and galectin-2 led to impaired perfusion restoration and a smaller average vessel diameter. Also, less macrophages were seen in the perivascular space, indicating impaired monocyte recruitment [92]. This suggests that blockade of blockade of galectin-2 could possibly stimulate arteriogenesis. 

## ARTERIOGENESIS VERSUS ATHEROSCLEROSIS

One of the pitfalls of stimulating arteriogenesis is that many of the mechanisms that induce collateral growth also contribute to the formation of atherosclerosis. This is of particular importance in patients who already suffer from narrowing coronaries. For the development of clinically usable therapies it is of vital importance that these agents do not enhance atherosclerosis, thereby cancelling their beneficial effects. For example, an increase in shear stress can induce arteriogenesis as well as atherosclerosis [93]. Local treatment with MCP-1 also had systemic effects, causing circulating monocytes to adhere at the aortic wall, an increase in total plaque surface area and a change in plaque composition [27, 94]. Besides this, some of the inhibitors of collateral vessel formation protect against atherosclerosis, as is the case with Cx37. Increased expression of Cx37 protects against atherosclerosis [84], but inhibits collateral growth [80, 95]. Interestingly, the secondary endothelial expressed connexin isoform, Cx40 has an atheroprotective effect as well as an arteriogenic effect [95, 96]. 

Both M1 and M2 macrophages are readily present in atherosclerotic plaques, although mainly M1 macrophages are associated with unstable plaques [94, 97]. Stimulating the accumulation of macrophages can thereby have unwanted consequences, but specific M2-macrophage recruitment may overcome this problem. IFN-β does possibly not have these unwanted side effects. Although IFN-β suppresses the atherogenic MMP-9 [98] It was recently shown that blocking of IFN-β has anti-atherogenic effects [99].

## DISCUSSION

On a global scale the prevalence of CAD is still rising [1]. In the past century a number of different therapies have been developed and especially since the 50’s and 60’s, when the first coronary catheterizations and CABG’s were performed, drastic changes have been witnessed. Not only an improvement in life-style but also due to evidence based medicine and innovative techniques and materials, mortality has declined constantly over the years. In industrialized counties, for most patients with CAD this means a significant increase in life span and improvement of quality of life. Unfortunately, not all CAD patients receive such efficient treatment methods. For example, physical condition or unsuccessful primary therapy can exclude some patients from interventional therapy. Also, in several parts of the world, facilities are not or only limited available to perform CABG or PCI. Therefore, pharmacological stimulation of arteriogenesis would be a very valuable addition to the currently available armamentarium of treating cardiologists worldwide. 

Although not fully understood yet, important progress has been made over the past 30 years in unraveling the mechanisms of collateral artery growth using the peripheral animal model. It involves growth factors, chemoattractants, degradation factors, survival factors and vasodilators. Several recently identified pro-arteriogenic targets are currently tested in preclinical models. It must be mentioned though that the translation from bench to bedside has been confronted with many difficulties. Data from animal studies using the hindlimb model is not easily transferred to the human coronary system.

It is now understood that apart from circulating monocytes, also tissue resident macrophages play a role in the formation of collateral arteries. The M2 macrophage is considered to be the most arteriogenic. Although native in normal tissue a rise in M2 concentration further propels vascular growth. Shifting the balance of M1/M2 macrophages is interesting from a clinical perspective. By stimulating the tissue resident cells to proliferate, the process of cell attraction need not be targeted. In this way vascular remodeling can possibly be initiated sooner. Further, the atherosclerotic ‘side effects’ might occur to a lesser extent. 

Genetic variation in the human capacity to form collateral arteries [100] seems to be steered mainly by a difference in expression of natural inhibitors of vascular proliferation [101, 102]. Monoclonal antibody therapy against such inhibiting factors (or their receptors) is another potential new strategy to induce collateral artery growth. As in many other mechanisms in the human body, arteriogenesis is a delicate balance between promoters and inhibitors and tipping this balance in a favorable direction is a big challenge still lying ahead. 

## Figures and Tables

**Table 1.** Known Markers for Human M1 and M2 Macrophages

M1 Macrophages	M2 Macrophages
CD40	Manose Receptor/CD206
CD86	Arginase-1
iNOS	RELM-alpha
CD16	YM1
MHCII	DCIR
CD32	CCL17
CXCL10	CCL13
CCL8	CCL14
CCL15	CCL23
CCL19	CCL26
CCL20	IL-4
IL-6	IL-33
IL-12	IL-10
IL-15	IL-13
IL-23	Fibronectin
CXCL9	CCL22
CXCL-10	
